# Supplementary material for: End Sequence Analysis Toolkit (ESAT) expands the extractable information from single-cell RNA-seq data
Source: Genome Res. 2016 Oct;26(10):1397–410. doi: 10.1101/gr.207902.116 (PMC5052061; doi:10.1101/gr.207902.116)
Supplement: Supplemental Material [file supp_gr.207902.116_Supp_Materials.tar.gz › ESAT-esat_v0.1/doc/html/classbroad_1_1core_1_1primer3_1_1_hairpin_kmer.html]

ESAT: broad.core.primer3.HairpinKmer Class Reference


ESAT


- Main Page
- Related Pages
- Packages
- Classes
- Files

- Class List
- Class Index
- Class Hierarchy
- Class Members

All Classes Namespaces Files Functions Variables Enumerator Pages

- broad
- core
- primer3
- HairpinKmer

Public Member Functions |
List of all members

broad.core.primer3.HairpinKmer Class Reference

Inheritance diagram for broad.core.primer3.HairpinKmer:

[legend]

Collaboration diagram for broad.core.primer3.HairpinKmer:

[legend]

|  |  |
| --- | --- |
| Public Member Functions | |
|  | HairpinKmer (String kmer, int relativeStart, int relativeEnd) |
|  | |
|  | HairpinKmer (String kmer, int relativeStart, int relativeEnd, Map< String, Double > mirLookup, Set< String > lookup2, Set< String > rcLookup2) |
|  | |
| void | addTranscript (RNAiGeneAnnotation transcript) |
|  | |
| Collection< RNAiGeneAnnotation > | getTranscripts () |
|  | |
| void | setPercentVariantsCovered (double percent) |
|  | |
| double | getPercentVariantsCovered () |
|  | |
| String | getKmerSequence () |
|  | |
| int | getKmerStartPosition () |
|  | |
| int | getKmerEndPosition () |
|  | |
| double | getRS8Score () |
|  | |
| double | getOriginalScore () |
|  | |
| double | getMirScore () |
|  | |
| int | compareTo (HairpinKmer kmer) |
|  | |
| boolean | setRS8Score (double score) |
|  | |
| boolean | setOriginalScore (double score) |
|  | |
| boolean | setMirScore (double score) |
|  | |
| void | setBlastHit (boolean b) |
|  | |
| boolean | getBlastHit () |
|  | |
| List< SequenceRegion > | getRelativePositions (RNAiGeneAnnotation transcript) throws SearchException |
|  | |
| int | getOverlap (HairpinKmer hp) |
|  | |
| double | getPercentGC () |
|  | |
| int | maxSelfComplementarity () |
|  | |

## Constructor & Destructor Documentation

|  |  |  |  |
| --- | --- | --- | --- |
| broad.core.primer3.HairpinKmer.HairpinKmer | ( | String | *kmer*, |
|  |  | int | *relativeStart*, |
|  |  | int | *relativeEnd* |
|  | ) |  |  |

|  |  |  |  |
| --- | --- | --- | --- |
| broad.core.primer3.HairpinKmer.HairpinKmer | ( | String | *kmer*, |
|  |  | int | *relativeStart*, |
|  |  | int | *relativeEnd*, |
|  |  | Map< String, Double > | *mirLookup*, |
|  |  | Set< String > | *lookup2*, |
|  |  | Set< String > | *rcLookup2* |
|  | ) |  |  |

## Member Function Documentation

|  |  |  |  |  |  |
| --- | --- | --- | --- | --- | --- |
| void broad.core.primer3.HairpinKmer.addTranscript | ( | RNAiGeneAnnotation | *transcript* | ) |  |

|  |  |  |  |  |  |
| --- | --- | --- | --- | --- | --- |
| int broad.core.primer3.HairpinKmer.compareTo | ( | HairpinKmer | *kmer* | ) |  |

Here is the call graph for this function:

|  |  |  |  |  |
| --- | --- | --- | --- | --- |
| boolean broad.core.primer3.HairpinKmer.getBlastHit | ( |  | ) |  |

|  |  |  |  |  |
| --- | --- | --- | --- | --- |
| int broad.core.primer3.HairpinKmer.getKmerEndPosition | ( |  | ) |  |

Here is the caller graph for this function:

|  |  |  |  |  |
| --- | --- | --- | --- | --- |
| String broad.core.primer3.HairpinKmer.getKmerSequence | ( |  | ) |  |

Here is the caller graph for this function:

|  |  |  |  |  |
| --- | --- | --- | --- | --- |
| int broad.core.primer3.HairpinKmer.getKmerStartPosition | ( |  | ) |  |

Here is the caller graph for this function:

|  |  |  |  |  |
| --- | --- | --- | --- | --- |
| double broad.core.primer3.HairpinKmer.getMirScore | ( |  | ) |  |

|  |  |  |  |  |
| --- | --- | --- | --- | --- |
| double broad.core.primer3.HairpinKmer.getOriginalScore | ( |  | ) |  |

|  |  |  |  |  |  |
| --- | --- | --- | --- | --- | --- |
| int broad.core.primer3.HairpinKmer.getOverlap | ( | HairpinKmer | *hp* | ) |  |

Here is the call graph for this function:

|  |  |  |  |  |
| --- | --- | --- | --- | --- |
| double broad.core.primer3.HairpinKmer.getPercentGC | ( |  | ) |  |

|  |  |  |  |  |
| --- | --- | --- | --- | --- |
| double broad.core.primer3.HairpinKmer.getPercentVariantsCovered | ( |  | ) |  |

Here is the caller graph for this function:

|  |  |  |  |  |  |
| --- | --- | --- | --- | --- | --- |
| List<SequenceRegion> broad.core.primer3.HairpinKmer.getRelativePositions | ( | RNAiGeneAnnotation | *transcript* | ) | throws SearchException |

|  |  |  |  |  |
| --- | --- | --- | --- | --- |
| double broad.core.primer3.HairpinKmer.getRS8Score | ( |  | ) |  |

Here is the caller graph for this function:

|  |  |  |  |  |
| --- | --- | --- | --- | --- |
| Collection<RNAiGeneAnnotation> broad.core.primer3.HairpinKmer.getTranscripts | ( |  | ) |  |

|  |  |  |  |  |
| --- | --- | --- | --- | --- |
| int broad.core.primer3.HairpinKmer.maxSelfComplementarity | ( |  | ) |  |

Here is the call graph for this function:

|  |  |  |  |  |  |
| --- | --- | --- | --- | --- | --- |
| void broad.core.primer3.HairpinKmer.setBlastHit | ( | boolean | *b* | ) |  |

|  |  |  |  |  |  |
| --- | --- | --- | --- | --- | --- |
| boolean broad.core.primer3.HairpinKmer.setMirScore | ( | double | *score* | ) |  |

|  |  |  |  |  |  |
| --- | --- | --- | --- | --- | --- |
| boolean broad.core.primer3.HairpinKmer.setOriginalScore | ( | double | *score* | ) |  |

|  |  |  |  |  |  |
| --- | --- | --- | --- | --- | --- |
| void broad.core.primer3.HairpinKmer.setPercentVariantsCovered | ( | double | *percent* | ) |  |

|  |  |  |  |  |  |
| --- | --- | --- | --- | --- | --- |
| boolean broad.core.primer3.HairpinKmer.setRS8Score | ( | double | *score* | ) |  |

---

The documentation for this class was generated from the following file:

- java/broad/core/primer3/HairpinKmer.java


---

Generated on Fri Aug 15 2014 15:10:55 for ESAT by  

 1.8.7
